# Dual mTORC1/C2 inhibitors: gerosuppressors with potential anti-aging effect

**DOI:** 10.18632/oncotarget.5563

**Published:** 2015-09-10

**Authors:** Pedro Sousa-Victor, Laura García-Prat, Pura Muñoz-Cánoves

**Affiliations:** ^1^ Buck Institute for Research on Aging, Novato, CA, USA; ^2^ Cell Biology Group, Department of Experimental and Health Sciences, Pompeu Fabra University (UPF), CIBER on Neurodegenerative Diseases (CIBERNED), and ICREA, Barcelona, Spain

**Keywords:** senescence, aging, quiescence, geroconversion, mTOR

## Abstract

Over the past decade, our understanding of the molecular and cellular mechanisms presiding over cellular and tissue decline with aging has greatly advanced. Classical hallmarks of aging cell include increasing levels of reactive oxygen species, DNA damage and senescence entry, which disrupt tissue architecture and function. Tissue dysfunction with aging has been shown to correlate with a cellular switch from a G0 reversible quiescence state into a G0 irreversible senescence state (geroconversion), causing a permanent proliferative block. The TOR (target of rapamycin) kinase has been shown to promote geroconversion. Rapamycin and other rapalogs specifically suppress activity of the mammalian TOR (mTOR) complex 1 (mTORC1) -but not mTOR complex 2 (mTORC2)- and decrease senescence entry, thus preserving proliferative potential. In this perspective, we briefly comment recent progress of Leontieva and colleagues showing a new class of non-rapalog drugs that target simultaneously mTORC1 and mTORC2 and prevent geroconversion in a more efficient way than rapamycin. Its potential future use as rejuvenating, anti-aging therapeutics is therefore proposed.

Aging is a nearly universal process affecting all tissues of an organism and can be defined as the accumulation of damage in molecules, cells and tissues over a lifetime. A hallmark of aging is alteration of organismal homeostasis and progressive decline of tissue functions [1, 2]. In addition, aging has been associated to cellular senescence [3]. However, the most striking and compelling prove of this link came from the van Deursen lab, whereby removal of senescent cells by genetic manipulation in a progeric mouse model delayed the aging phenotype and related disease [4]. Senescent cells are dysfunctional cells that are permanently withdrawn from the cell cycle and have ceased proliferation [5]. This is distinct to the reversible cell cycle arrest of quiescent cells, which can maintain re-proliferative potential (RP) [6]. Recently, stem cells of skeletal muscle from aged mice have been shown to lose their normal reversible quiescence state and acquire an irreversible senescent state, correlating with an age-associated tissue regenerative decline. This demonstrated an age associated quiescence-to-senescence transition in physiological aging. Furthermore, this supported that promoting cell quiescence in aging, or preventing senescence, may constitute potential anti-aging strategies for the increasing elderly human population. In cultured cells, this quiescence-to-senescence transition leading to gerogenic conversion is known as geroconversion [7].

Although no single hallmark can be used to define senescence, this state is characterized by cellular hypertrophy, SA-β-Gal staining and hyper-secretory phenotypes [8, 9]. Senescence is thus a driving force of age-related pathologies by both acting on stem cells to limit tissues regenerative potential and through the secretory role in a pro-inflammatory condition known as inflammaging [10]. Understanding the mechanisms involved in establishing the senescent arrest may help design strategies to delay or reverse age-dependent loss of tissue function and regenerative capacity. Work from the Blagosklonny group has shown that in arrested cells, geroconversion depends on mTOR [7, 11]. Observations that cellular hypertrophy in senescent cells in culture was a consequence of mTOR activity and that inhibiting mTOR allowed for the preservation of RP in the presence of a cell cycle arrest established a close link between growth pathways and senescence. Indeed, in cells arrested by the CDK inhibitors p16 and p21, mTOR inhibitor rapamycin decelerates the cell gerogenic conversion (geroconversion) to senescence [11]. Thus, mTOR pathway inhibition appears as a possible gerosuppressing (anti-aging) strategy.

In this issue of Oncotarget, Leontieva et al show that this concept is extensive to a new class of non-rapalog drugs that target simultaneously mTORC1 and mTORC2. Torin1 and PP242 are ATP-competitive inhibitors of mTOR kinase activity and thus inhibit the function of both complexes [12]. The authors show a dose-dependent effect of these drugs in preventing geroconversion of cells in culture. In the experiments reported senescence was induced by three different methods in three different cellular contexts - (1) p21-expression in HT1080 human cells; (2) etoposide/doxorubicin treatment of WI38t human fibroblasts and (3) PMA treatment of SKBR3 breast adenocarcinoma cell line. In all cases cell cycle arrest leads to geroconversion, as measured by the lack of re-proliferating capacity upon withdrawal of the senescence stimulus. Importantly, treatment with Torin1 or PP242 prevented senescence phenotypes and geroconversion, correlating with inhibition of mTOR activity. Interestingly, Torin1 and PP242 were more efficient at suppressing the senescent morphology and hypertrophy than rapamycin, suggesting that these cell characteristics partially depend on the rapamycin-insensitive functions of mTOR [13].

Several studies indicate that the link between mTOR signaling and aging and longevity is conserved across species [14]. While rapamycin has now extensive experimental support as a potential anti-aging intervention in mammals [15], the use of dual mTOR inhibitors will likely carry increased potential for side effects. However, the demonstration that pan-mTOR inhibitors at low concentrations are capable of suppressing geroconversion and preserving RP [13] reinforces their potential future use as anti-aging therapeutics.

Since geroconversion is a phenomena that occurs to stem cells *in vivo* during physiological aging [16, 17], and because *in vivo* elimination of senescent cells delayed aging and age-associated diseases [4], exploration of a larger variety of gerosuppressive drugs (such as mTOR inhibitors) can contribute to the development of rejuvenation strategies.

**Figure 1:**
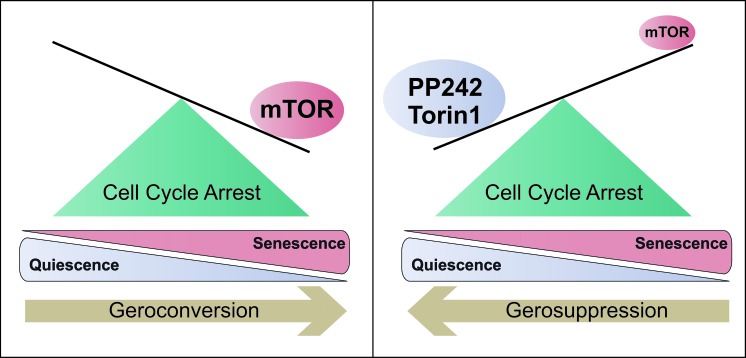
Quiescence vs. Senescence In the G0 phase of the cell cycle, mTOR levels determine cell cycle reversibility. High levels of mTOR drive cells to an irreversible senescence state (geroconversion), while mTOR inhibition by rapalogs, such as Torin1 and PP242, maintain cells in the quiescence state and preserve their re-proliferative potential (gerosuppression).

## References

[1] Lopez-Otin C (2013). The hallmarks of aging. Cell.

[2] Madaro L, Latella L (2015). Forever young: rejuvenating muscle satellite cells. Front Aging Neurosci.

[3] van Deursen JM (2014). The role of senescent cells in ageing. Nature.

[4] Baker DJ (2011). Clearance of p16Ink4a-positive senescent cells delays ageing-associated disorders. Nature.

[5] Campisi J (2011). Cellular senescence: putting the paradoxes in perspective. Curr Opin Genet Dev.

[6] Blagosklonny MV (2011). Cell cycle arrest is not senescence. Aging (Albany NY).

[7] Blagosklonny MV (2014). Geroconversion: irreversible step to cellular senescence. Cell Cycle.

[8] Campisi J, d'Adda di Fagagna F (2007). Cellular senescence: when bad things happen to good cells. Nat Rev Mol Cell Biol.

[9] Kuilman T (2010). The essence of senescence. Genes Dev.

[10] Campisi J, Robert L (2014). Cell senescence: role in aging and age-related diseases. Interdiscip Top Gerontol.

[11] Demidenko ZN (2009). Rapamycin decelerates cellular senescence. Cell Cycle.

[12] Benjamin D (2011). Rapamycin passes the torch: a new generation of mTOR inhibitors. Nat Rev Drug Discov.

[13] Leontieva OV, Demidenko ZN, Blagosklonny MV (2015). Dual mTORC1/C2 inhibitors suppress cellular geroconversion (a senescence program). Oncotarget.

[14] Johnson SC, Rabinovitch PS, Kaeberlein M (2013). mTOR is a key modulator of ageing and age-related disease. Nature.

[15] Blagosklonny MV (2010). Why human lifespan is rapidly increasing: solving “longevity riddle” with “revealed-slow-aging” hypothesis. Aging (Albany NY).

[16] Sousa-Victor P (2014). Geriatric muscle stem cells switch reversible quiescence into senescence. Nature.

[17] Sousa-Victor P, Perdiguero E, Munoz-Canoves P (2014). Geroconversion of aged muscle stem cells under regenerative pressure. Cell Cycle.

